# Daily Social Interactions as a Route to Purposeful Days in Older Adulthood

**DOI:** 10.1093/geroni/igab046.820

**Published:** 2021-12-17

**Authors:** Gabrielle Pfund, Mathias Allemand, Matthias Hofer

**Affiliations:** 1 Washington University in St. Louis, Saint Louis, Missouri, United States; 2 University of Zurich, Zurich, Zurich, Switzerland

## Abstract

Sense of purpose predicts slower cognitive decline, reduced risk for health issues, and greater longevity (Pfund & Lewis, 2020). However, work is limited regarding how we can help older adults maintain purposefulness in daily life. The current study explored positive daily social interactions as a route to daily purposefulness in older adults, using a measurement burst design. Older adults completed surveys for five-day bursts spread six months apart (Mean age = 70.75, SD = 7.23; n = 104). Multilevel models demonstrated that on days when individuals reported more positive social interactions, they reported feeling more purposeful (b = 0.39, 95% CI [0.28, 0.51]) when accounting for health, employment, and relationship status. Employment status moderated this association, as daily social interactions were more strongly associated with daily purpose for unemployed/retired individuals (b = -0.23, 95% CI [-0.38, -0.08]). Positive social interactions thus may help older adults maintain purposefulness, particularly after retirement.

